# Characterizing and explaining the impact of disease-associated mutations in proteins without known structures or structural homologs

**DOI:** 10.1093/bib/bbac187

**Published:** 2022-06-01

**Authors:** Neeladri Sen, Ivan Anishchenko, Nicola Bordin, Ian Sillitoe, Sameer Velankar, David Baker, Christine Orengo

**Affiliations:** Institute of Structural and Molecular Biology, University College London, London, WC1E 6BT, UK; Department of Biochemistry, University of Washington, Seattle, WA 98195, USA; Institute for Protein Design, University of Washington, Seattle, WA 98195, USA; Institute of Structural and Molecular Biology, University College London, London, WC1E 6BT, UK; Institute of Structural and Molecular Biology, University College London, London, WC1E 6BT, UK; Protein Data Bank in Europe, European Molecular Biology Laboratory, European Bioinformatics Institute (EMBL-EBI), Wellcome Genome Campus, Hinxton, Cambridge CB10 1SD, UK; Department of Biochemistry, University of Washington, Seattle, WA 98195, USA; Institute for Protein Design, University of Washington, Seattle, WA 98195, USA; Howard Hughes Medical Institute, University of Washington, Seattle, WA 98195, USA; Institute of Structural and Molecular Biology, University College London, London, WC1E 6BT, UK

**Keywords:** protein structure modeling, mutation, AlphaFold, RoseTTAFold, disease-associated, functional site

## Abstract

Mutations in human proteins lead to diseases. The structure of these proteins can help understand the mechanism of such diseases and develop therapeutics against them. With improved deep learning techniques, such as RoseTTAFold and AlphaFold, we can predict the structure of proteins even in the absence of structural homologs. We modeled and extracted the domains from 553 disease-associated human proteins without known protein structures or close homologs in the Protein Databank. We noticed that the model quality was higher and the Root mean square deviation (RMSD) lower between AlphaFold and RoseTTAFold models for domains that could be assigned to CATH families as compared to those which could only be assigned to Pfam families of unknown structure or could not be assigned to either. We predicted ligand-binding sites, protein–protein interfaces and conserved residues in these predicted structures. We then explored whether the disease-associated missense mutations were in the proximity of these predicted functional sites, whether they destabilized the protein structure based on ddG calculations or whether they were predicted to be pathogenic. We could explain 80% of these disease-associated mutations based on proximity to functional sites, structural destabilization or pathogenicity. When compared to polymorphisms, a larger percentage of disease-associated missense mutations were buried, closer to predicted functional sites, predicted as destabilizing and pathogenic. Usage of models from the two state-of-the-art techniques provide better confidence in our predictions, and we explain 93 additional mutations based on RoseTTAFold models which could not be explained based solely on AlphaFold models.

## Introduction

Mutations in genomes can either be in the coding regions or noncoding regions. Mutations in the noncoding regions of the genome largely affect protein production by initiating it at the wrong time and wrong place or reduce and eliminate the production of proteins. These lead to various diseases, such as congenital heart diseases, pancreatic agenesis, campomelic dysplasia, developmental disorders, and are very commonly found in cancer [1–4]. A missense mutation (change in the amino acid to another) in the coding region of the genome can lead to changes in or near catalytic site residues [5, 6], ligand-binding sites [7, 8], protein–protein interface sites [9, 10], allosteric sites [11], etc., modifying the protein or rendering it inactive. The sequence of amino acids dictates the structure of the protein [12]. Some missense mutations can hence lead to changes in the structure of the protein [13, 14], which make the protein non-functional.

Single nucleotide polymorphisms (SNPs) are common in genes, some of which lead to disease and are being collated in databases such as dbSNP [15] and the 1000 genomes project [16]. Databases such as ClinVar [17] link mutations in humans with phenotypes. Specific databases such as COSMIC [18], OncoVar [19] and DoCM [20] contain information on cancer-related mutations. Several other specialized databases containing subsets of these mutations exist, such as KinMutBase (for human disease-associated protein kinase mutations) [21] and ActiveDriverDB (mutations at protein post-translational modification sites), [22] among others. UniProt-KB [23] and PDBe-KB [24] also contain experimental and computational annotations of mutations and associated phenotypes. Humsavar [23] is a database of human variants from literature reports and therefore tends to have more substantial annotations from experimental evidence. DBSAV [25] is a database that contains all SNPs in the human proteome and a predicted score of their deleteriousness.

Protein structures can help gain insights into the mechanism of these disease-associated mutations. However, experimental determination of protein structures is time-consuming, expensive and difficult. With the increase in the size of proteins and complexes, it becomes increasingly challenging to experimentally determine their structures. Hence, computational models can help in such cases. Homology modeling techniques (such as MODELLER [26, 27], SWISS-MODEL [28], etc.) can be used to model sequences in the presence of structural homologs having sequence identity ≥30%, but a large number of protein sequences have no close homologs. *Ab initio* protein structure modeling techniques (ROSETTA [29], I-TASSER [30], etc.) are used in the absence of structural homologs. More recently, deep neural networks trained on the massive body of available protein sequence and structure data have been shown to significantly outperform traditional homology modeling or *ab initio* approaches in terms of the accuracy of the resulting model (e.g. RaptorX [31], DMPFold [32], trRosetta [33], AlphaFold.v1 [34], etc.). Models, such as AlphaFold.v2 [35] and RoseTTAFold [36], trained end to end to directly predict 3D model coordinates were shown to be able to reach near-experimental accuracy. AlphaFold was shown to be the best-performing technique for protein structure prediction in CASP14 (https://predictioncenter.org/casp14/). In July 2021, AlphaFold released the structures of the entire reference proteome of 21 organisms [37], which had about 50% of the residues (that could not be modeled using homology) predicted with confidence [38]. Recent studies have shown that these high-quality models can be used to predict binding sites and effects of mutations and these calculations and predictions are similar to those obtained from experiments or experimental structures [38].

Along with the protein structure, experimental and computational determination of functional residues can help explain the effect of mutations on proteins. Mutations on or near functional sites are likely to affect the functioning of the proteins. The current strategies for the computational prediction of functional sites such as catalytic sites, ligand-binding sites, allosteric sites, protein–protein interaction sites, etc. have been reviewed elsewhere [39–42]. In addition to the predictions of functional sites, the energetic effect of mutations can also be calculated using various tools [43–46], which determine the free energy change of the mutation. These tools can predict whether the mutation will destabilize the protein structure, possibly affecting its functioning. The likely deleteriousness of these mutations can be calculated based on various machine learning-based pathogenicity predictors, such as CADD, EVE, MutPred2, etc. [47–49].

Homology models have been previously used to explain disease-associated mutations [50, 51]. In this article, we model and exploit RoseTTAFold and AlphaFold models of human proteins without known protein structures or homologs in the Protein Databank (PDB) [52] (sequence identity < 30%) and explain the effect of deleterious missense mutations by checking if these mutations are on or near a predicted functional site, conserved site, lead to protein structure destabilization or are pathogenic (mutations which might lead to disease).

## Results

### Protein domains for disease-associated proteins

For our analysis, we considered 553 human disease-associated proteins without known protein structures or close homologs in the PDB (see the Curation of disease-associated human proteins section for criteria used to identify close homologs). These proteins were selected from the VarSite [53] database, containing disease site information from Uniprot, ClinVar and gnomAD (see Curation of disease-associated human proteins section for further details on protein selection). We checked the PANTHER [54–56] protein classes these proteins fall into. Of the 553 proteins, only 243 proteins have been associated with 20 out of 24 PANTHER classes. The most highly represented protein classes are metabolite interconverting proteins, transporters and scaffold/adaptor proteins, containing 71, 36 and 16 proteins, respectively (Supplementary Table 2 available online at http://bib.oxfordjournals.org/). These are followed by chaperons, protein-modifying enzymes and membrane traffic proteins containing 13 proteins each. We performed gene ontology analysis using PANTHER, which showed the enrichment of terms that can be associated with transport or metabolite interconverting enzymes (details about gene ontology are given in Supplementary Text 1 available online at http://bib.oxfordjournals.org/). However, we would like to point out that the dataset is somewhat biased in the sense that these proteins are those without any homologs in the PDB. It is possible that many of them are therefore difficult to crystallize.

**Figure 1:**
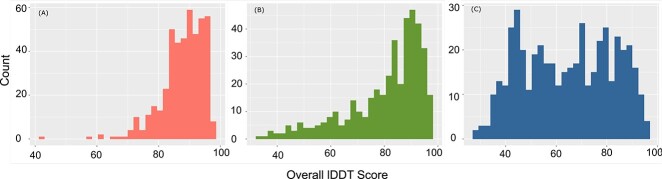
The model quality of AlphaFold models for (**A**) CATH domains (**B**) Pfam domains and (**C**) unassigned domains.

Out of the 553 proteins, regions in 198 proteins mapped to CATH. Of the remaining proteins or regions of proteins not mapped to CATH, 341 mapped to Pfam, leaving 14 proteins that could not be assigned to CATH or Pfam domains. CATH [57, 58] and Pfam [59] superfamilies can be functionally diverse, with 62% of the protein space being covered by the largest 200 CATH superfamilies [60]. Hence, only some of the members of these superfamilies will function similarly, requiring the need to subclassify them into functionally similar families called FunFams [61] (Supplementary Text 11 available online at http://bib.oxfordjournals.org/). The 198 proteins assigned to CATH comprised 309 domains which were assigned to 297 FunFams and 117 CATH superfamilies. Rossman and immunoglobulin folds were among the most represented in the dataset (Supplementary Figure 1 available online at http://bib.oxfordjournals.org/). This is not surprising as these folds are among the most commonly occurring folds in the CATH database and appear to be adopted by a large proportion of UniProt sequences [62, 63] (refer Supplementary Text 9 and Supplementary Figure 2, available online at http://bib.oxfordjournals.org/, for details about model quality across superfamily). The remaining proteins and regions which were not assigned to CATH domains were scanned against Pfam FunFams. 416 domains belonging to 341 proteins were matched to 376 unique Pfam FunFams. A total of 496 regions of proteins (at least 50-residue-long stretches) belonging to 332 proteins were designated as unassigned domains that did not map to CATH/Pfam domains. Some of these (41) could be putatively assigned to CATH superfamilies using more advanced sequence search strategies and structure comparisons using the predicted structure (see Supplementary Text 10 available online at http://bib.oxfordjournals.org/) [64, 65].

### Comparison of domain models generated using RoseTTAFold and AlphaFold

The quality of the models was assessed using the average predicted lDDT score [66] (Supplementary Text 11, available online at http://bib.oxfordjournals.org/, for details about lDDT). The pLDDT value reported by RoseTTAfold can range from 0 to 1, while for AlphaFold2, the range is between 0 and 100. Therefore, 0.7 for RoseTTAfold models is equivalent to 70 for AlphaFold2 models. RoseTTAFold models with a score of 0.7 and AlphaFold models with a score of 70 were considered as good models, as recommended by the developers of these techniques. Furthermore, 304/309 (98%), 328/416 (79%) and 198/469 (42%) CATH, Pfam and unassigned domains, respectively, were good AlphaFold models (Figure 1). However, for the RoseTTAFold models 274/309 (89%), 240/416 (58%) and 79/469 (17%) CATH, Pfam and unassigned domains, respectively, were good models. Thus 72% AlphaFold models and 58% RoseTTAFold models were of good quality.

We did not have access to the multiple sequence alignments (MSAs) used to build the AlphaFold models; hence, we analyzed the MSAs used to build the RoseTTAFold models. We calculated the number of effective sequences (Neff), diversity of positions (DOPs), percent scorecons and number of taxons in the alignment to check the information content of the MSA used to build the models [67]. Neff provides an estimate of the number of sequence clusters at 80% sequence identity, thereby providing a measure of the diversity of the dataset. Scorecons provide a conservation score for each of the positions in the MSA. Percent scorecons provide an estimate of the percentage of the conserved positions in the MSA, which also reflects the diversity of sequences in the MSA. Similarly, DOPs also provides an estimate of the diversity of the dataset and associated MSA based on conservation scores (see Supplementary Text 11, available online at http://bib.oxfordjournals.org/, for more details about each of the metrics). These measures quantify the diversity of the sequences in the alignment and not the quality of the alignment. Neff and the number of taxons in the alignment are higher for CATH models compared with the other two groups providing more coevolutionary information for those models (Supplementary Figure 3 available online at http://bib.oxfordjournals.org/). However, no clear correlation was found between Neff, DOPs, percent scorecons, number of taxons in the alignment and model quality (Supplementary Figure 4 available online at http://bib.oxfordjournals.org/). This trend is similar to that reported by AlphaFold and RosettaFold (refer details in Supplementary Text 2 available online at http://bib.oxfordjournals.org/) [35, 36].

**Figure 2:**
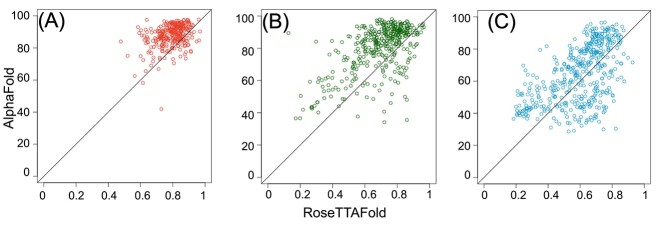
Model quality of AlphaFold models versus RoseTTAFold models for (**A**) CATH domains (**B**) Pfam domains and (**C**) unassigned domains.

Though the AlphaFold model quality was higher for 89% of the models compared to RoseTTAFold, 2, 27 and 45 CATH, Pfam and unassigned domains, respectively, had good RoseTTAFold models where the AlphaFold models were not of good quality (Figure 2). It should also be noted that the AlphaFold domains were extracted from the full proteins; however, RoseTTAFold models were built directly from the domain sequences. Residues in AlphaFold domains were modeled in the presence of the residues from other interacting domains, which was not the case for RoseTTAFold. Hence, added constraints introduced in the alignment and placement of residues with respect to neighbors from surrounding domains during modeling might have led to the better model quality of the AlphaFold models as compared to RoseTTAFold models.

**Figure 3:**
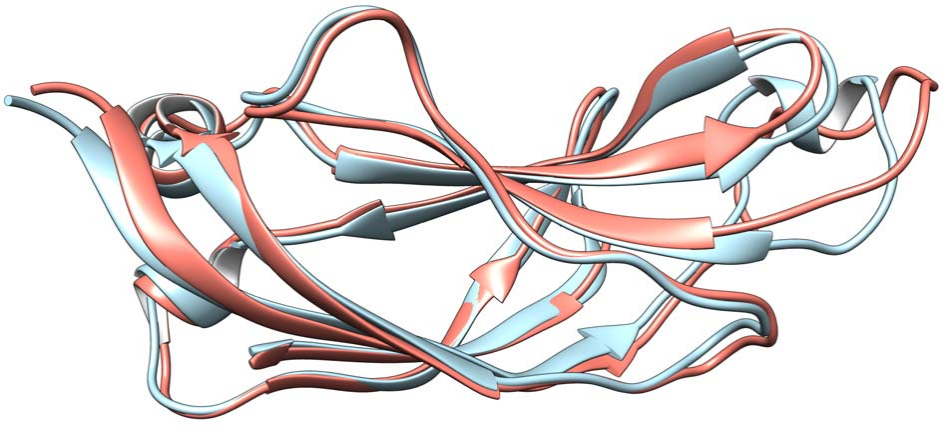
Ribbon model of superimposition of AlphaFold model (salmon) and RoseTTAFold model (sky blue) for CATH domain of C2 domain-containing protein 3, having an RMSD of 1 Å. Both the models are of good quality. Figure generated using CHIMERA [121].

**Figure 4:**
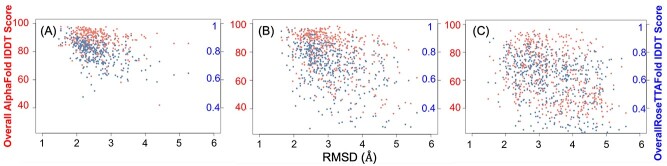
Distribution of model quality (AlphaFold in red, RoseTTAFold in blue) against the RMSD between the models for (**A**) CATH domains (**B**) Pfam domains and (**C**) unassigned domains.

Around 88%, 74% and 64% of the CATH, Pfam and unassigned domains had an RMSD < 2 Å between domains that had both good RoseTTAFold and AlphaFold models, indicating higher structural similarity for the CATH and Pfam domains for good-quality models (Supplementary Figure 5 available online at http://bib.oxfordjournals.org/). In some cases, simple orientation changes of helices or sheets with respect to one another in RoseTTAFold domain models would help in better superimposition than those of their AlphaFold counterparts, hence reducing the RMSD. Some of the models, including regions of the C2 domain-containing protein 3 (Uniprot Q4AC94) mapped onto CATH FunFams, showed RMSD < 1A between RoseTTAFold and AlphaFold models (Figure 3). As expected, the RMSD of the domains increased with a decrease in model quality (Figure 4). The correlation of RMSD with model quality is as expected and provides confidence in the techniques and their independence. The length of the model did not affect the model quality of AlphaFold models (correlation coefficient between length and model quality of 0.16, −0.13 and −0.03 for CATH, Pfam and unassigned domains) (Supplementary Figure 6 available online at http://bib.oxfordjournals.org/). However, the model quality was lower for longer Pfam and unassigned RoseTTAFold domain models (correlation coefficient between length and model quality of 0, −0.35 and −0.52 for CATH, Pfam and unassigned domains, respectively) (Supplementary Figure 6 available online at http://bib.oxfordjournals.org/).

### Disorder in protein domains

**Figure 5:**
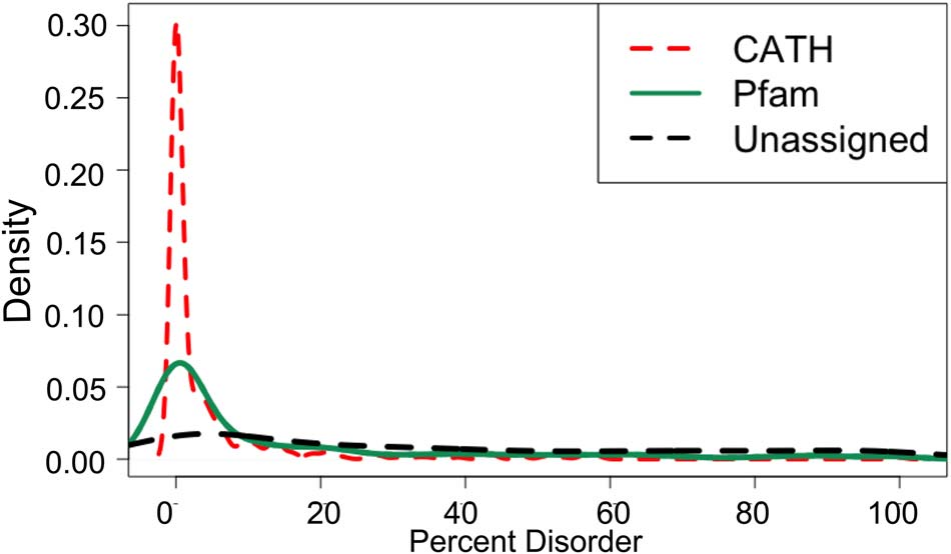
Density plot showing the distribution of percent disorder of the domain sequence for CATH (in red), Pfam (in green) and unassigned (in blue) domains.

The probability of a residue in the sequence to be disordered for the CATH, Pfam and unassigned domains was calculated using IUPred2A [68]. We then calculated the percentage of the domain sequence that is disordered. For the CATH, Pfam and unassigned domains, around 3%, 15% and 38% of the sequences, respectively, had greater than 40% of its sequence disordered (Figure 5). This indicates that the CATH domains had much lower disorder compared to Pfam which in turn was much lower compared with the unassigned domains. Given the fact that the disordered regions lack proper structure, the model quality of those regions will be low, hence lowering the overall domain model quality. The percentage of the predicted disordered region in the sequences of the unassigned domains had an inverse correlation coefficient of 0.74 and 0.41 with model quality for the AlphaFold and RoseTTAFold models, respectively (Supplementary Figure 7 available online at http://bib.oxfordjournals.org/), indicating that the model quality was lower because the sequences might be disordered. A low model quality for disordered regions provides higher confidence in the model quality scores.

### Characterization of disease-associated mutations

The total number of disease-associated missense mutations for our 553 modeled proteins in humsavar is 1730, corresponding to 1568 residue positions. Around 81% of the proteins in our dataset had 1 disease annotation for all the mutations. Forty of the proteins had >10 disease-associated mutations (Supplementary Figure 8**A** available online at http://bib.oxfordjournals.org/) of which 19 proteins had a single-disease annotation (Supplementary Figure 8**B** available online at http://bib.oxfordjournals.org/). For details, see Supplementary Text 3 available online at http://bib.oxfordjournals.org/. About 78% of the residues involving mutations leading to the same disease are structural neighbors (within 5 Å), suggesting a similar mechanism. The disease annotations in our dataset were mostly nervous system disorders followed by musculoskeletal system disorders according to the Human Disease Ontology database [69] (Supplementary Figure 9 and Supplementary Text 3 available online at http://bib.oxfordjournals.org/).

We checked if any of the mutations corresponded to gain of function (GOF)/loss of function (LOF) from the GOF/LOF database [70] based on the Human Gene Mutation database [71] and MAVEDB [72]. GOF and cancer mutations are usually clustered together in 3D space [73–75]. Thirteen out of 14 GOF mutations in the GOF/LOF database involve structural neighbors, whereas MAVEDB had no proteins from our dataset (Supplementary Text 3 available online at http://bib.oxfordjournals.org/).

We examined the relative abundance of the amino acids that the residues mutated into against proteins in the SwissProt51 database using Composition Profiler [76]. We noticed an enrichment of Cys, His, Pro, Arg, Ser and Trp with *P*-value < 0.05 (Supplementary Figure 10 and Supplementary Table 1 available online at http://bib.oxfordjournals.org/). These amino acids are either neutral polar or basic polar. In addition, Trp is a bulky amino acid and mutation into Trp can structurally destabilize the protein. Mutation into Cys might introduce unfavorable disulfide bonds and Pro can disrupt helical structures. These propensities are similar to a much larger amino acid mutation database study [77] for human disease mutations.

### Explaining disease-associated mutations

We checked if these mutations were on or near a predicted ligand-binding site (by P2Rank) or protein–protein interface (by meta-PPISP) or are conserved (by scorecons) in the FunFam or superfamily. Although there are several servers and tools for predicting ligand-binding sites and protein–protein interfaces that we recently reviewed [41], we used meta-PPISP and P2Rank based on availability, ease of installation, ability to run the scripts on all the protein domains and usage in recent studies (for details about choice of tools, see Supplementary Text 4 available online at http://bib.oxfordjournals.org/) [24, 37, 78–82].

We considered the residues within a 5 Å radius (heavy atom distance) (see Supplementary Text 4, available online at http://bib.oxfordjournals.org/, for the explanation of this threshold) [83–85] of a predicted functional site because mutations in those can lead to a change in the environment of the functional site residue, potentially leading to a modification or loss of activity. We limited our analysis to the structure of the wild-type protein as we were unsure of the ability of the techniques to build the mutant models. We also calculated the effect of the mutations on the stability of the protein using FoldX and DynaMut2 and pathogenicity of mutations using MutPred2.

**Figure 6:**
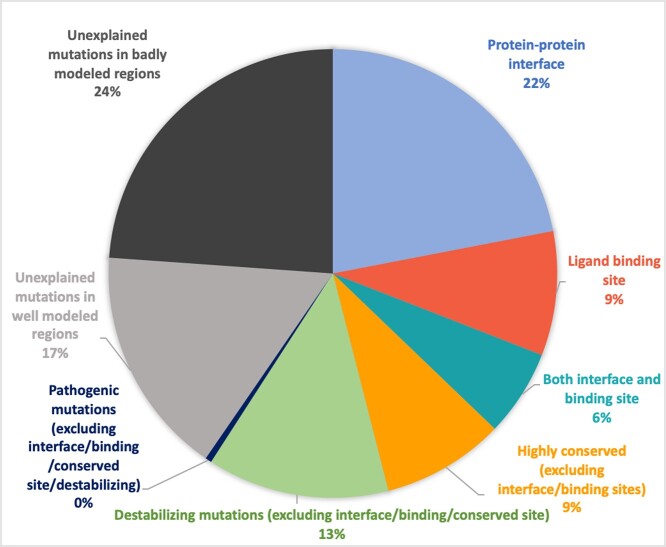
Percentage of disease-associated mutations that are predicted to be in or near a ligand-binding/protein–protein interface/conserved site in the AlphaFold domain models. Some of these mutations are predicted to be destabilizing or pathogenic.

Out of the 1568 disease-associated residue positions, 1317 residue positions (corresponding to 1389 mutations) are in high-confidence AlphaFold-modeled domains and residues (lDDT > 70). We did not analyze residues with lDDT < 70 as those are low-confidence regions of the models and hence should be treated with caution. For these domains, 122 CATH (585 residue positions), 143 Pfam (508 residue positions) and 61 unassigned domains (224 residue positions) had disease-associated mutations.

796 of these disease-associated residue positions (corresponding to 919 mutations) are either in or near a predicted functional site (Figure 6). In addition, 272 mutations (corresponding to 227 residue positions) were predicted to be destabilizing using FoldX (Figure 5). Other than these, eight mutations (corresponding to eight residue positions) were predicted to be pathogenic. Of all the disease-associated residue positions, 488 were near the predicted protein–protein interface, 163 near the predicted ligand-binding sites and 473 were near conserved sites. Furthermore, 816 mutations (corresponding to 689 residue positions) were predicted to be destabilizing by FoldX (ddG > 1) (refer Supplementary Figure 11 available online at http://bib.oxfordjournals.org/; Supplementary Text 4, available online at http://bib.oxfordjournals.org/, for the explanation of this threshold). We used two independent methods, FoldX and DynaMut2, to predict the free energy change of mutation. We found that for about 95% of the mutations predicted to have an impact by FoldX, DynaMut2 [86] also predicted impact, giving higher confidence in the predictions (Supplementary Figure 11 available online at http://bib.oxfordjournals.org/). We have not used the computed ddG value to quantify the impact of the mutation as such computed values might not correspond to actual ddG values. Moreover, 899 mutations (corresponding to 844 residue positions) were predicted as pathogenic using MutPred2. Of these, 638 mutations (corresponding to 629 proteins) were in the functional sites. In total, this suggests that we can provide some rationale for 78% of the disease-associated mutations in the well-modeled regions.

We calculated the solvent accessibility of residues whose mutations lead to disease and compared them against the distribution of the solvent accessibility of residues that make up the entire domain (Supplementary Figure 19 available online at http://bib.oxfordjournals.org/). We noticed that 42% (547 residues) of the residues that lead to disease were buried (residues with solvent accessibility <20 were considered buried [87]). This is much higher than the 21% of residues buried in the entire domain. A large number of the disease-associated mutations are buried, which could cause destabilization of the protein. Out of the 547 buried residues, 428 residues had mutations that were predicted destabilizing according to FoldX.

**Figure 7:**
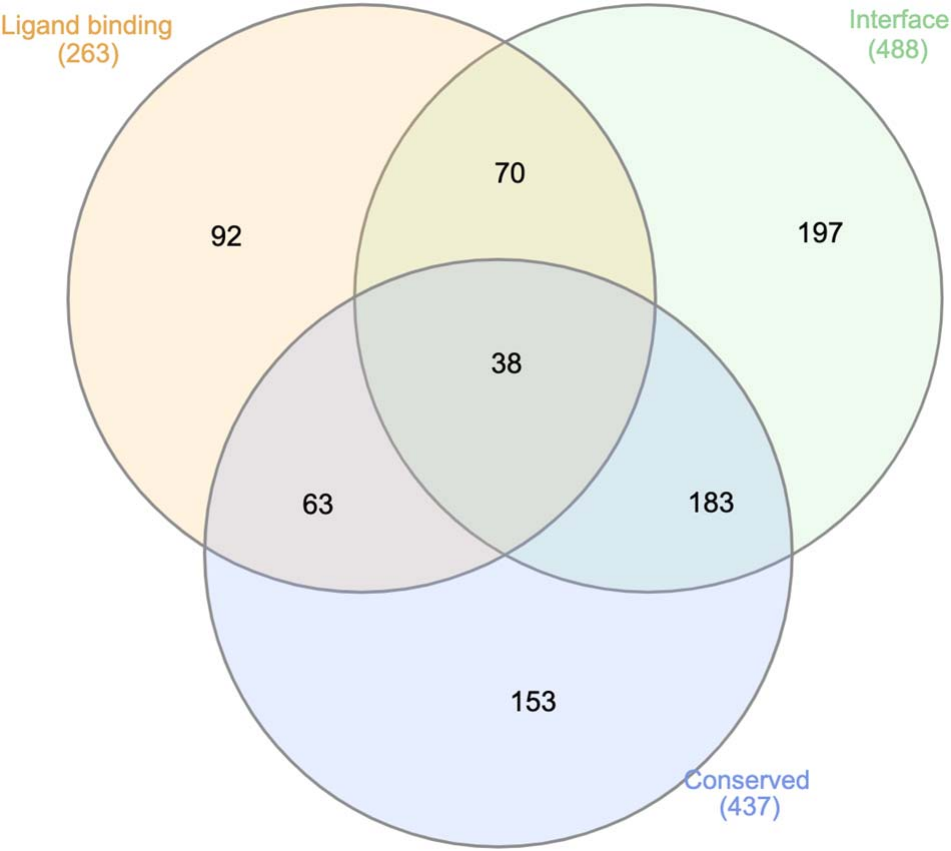
The overlap between the number of disease-associated mutation sites that are either predicted to be on or near a ligand-binding/protein–protein interface/conserved for the AlphaFold domain models. Figure generated using InteractiVenn [120].

When we consider whether mutations fall on overlapping functional sites, we observe that 108 mutations were predicted as both near a ligand-binding site and a protein–protein interface, 101 mutations either near a conserved residue or a ligand-binding site and 221 mutations were either near an interface or a conserved site (Figure 7). Around 44% of these mutations have multiple evidence for it lying on or near a functional site, which provides higher confidence in these sites having functional significance.

In addition, RoseTTAFold helped to find 93 additional mutations (in 44 proteins) near functional domain regions, which could not be explained using AlphaFold. The AlphaFold and RosettaFold models were very structurally similar to each other with an RMSD of 1.7+/− 0.6 Å (Supplementary Figure 12 available online at http://bib.oxfordjournals.org/). Small changes in orientations of the parts of the models led to low quality for AlphaFold model or prediction of functional site or larger distance of the functional site from mutations (Supplementary Figure 13 and Supplementary Text 8 available online at http://bib.oxfordjournals.org/). There was an overlap of 492 mutations predicted to be a functional site between the predictions derived from RoseTTAFold and AlphaFold models, providing higher confidence in these predictions.

**Figure 8:**
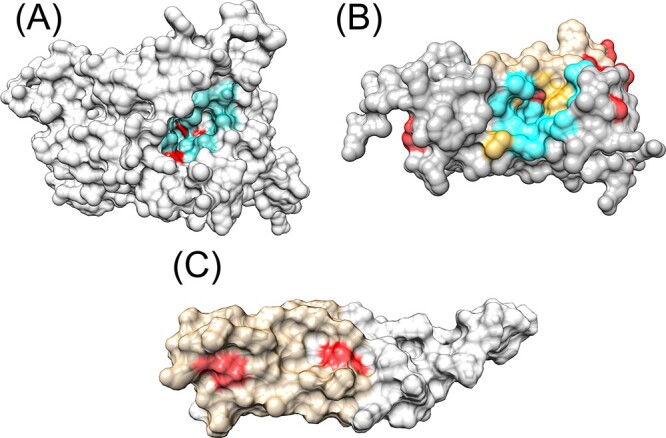
(**A**) AlphaFold models in gray surface representations for putative sodium-coupled neutral amino acid transporter 8, the residues in cyan are the predicted ligand-binding residues and in red are the disease-associated mutations. (**B**) AlphaFold models in gray surface representations for vitamin K epoxide reductase complex subunit 1, the residues in cyan are the predicted ligand-binding residues, tan are the predicted interface residues, golden are the residues predicted both as ligand binding and interface and in red are the disease-associated mutations. (**C**) RoseTTAFold model of early response-3 interacting protein-1 in gray surface representation. The predicted interface is in tan, and the disease-associated mutations are in red.

Mutations in residue numbers 32,33,233,236 and 412 in transmembrane helices of putative sodium-coupled neutral amino acid transporter 8 (Uniprot A6NNN8; Gene SLC38A8) lead to foveal hypoplasia 2. Other than residue 412, the others are spatially close to each other and are predicted either to be in or near a ligand-binding site. In addition, residue 233 is also predicted to be near a conserved site (Figure 8**A**). Mutations in the vitamin K epoxide reductase complex subunit 1 (Uniprot Q9BQB6; Gene VKORC1) lead to Coumarin resistance, which lie on or near the predicted ligand-binding sites and interface residues (Figure 8**B**). In another example, the immediate early response-3 interacting protein-1 (Uniprot Q9Y5U9, Gene IER3IP1) RoseTTAFold models had acceptable model quality, while the AlphaFold model was of low quality or the residue of interest was of low quality. Mutations in residue numbers 21 and 78 (associated with microcephaly, epilepsy and diabetes syndrome) are on the predicted protein–protein interface site (Figure 8**C**). In certain cases, such as the Arg300Cys mutation in beta-1,4 N-acetylgalactosaminyltransferase 1 (Uniprot Q00973; leading to spastic paraplegia 26, autosomal recessive) and the Ala241Thr mutation in feline leukemia virus subgroup C receptor-related protein 1 (Uniprot Q9Y5Y0) (leading to posterior column ataxia with retinitis pigmentosa), the impacts of the mutations could only be explained by large changes in ddG of the mutation (>2.5) (Supplementary Figure 14 available online at http://bib.oxfordjournals.org/). These residue positions were not close to any functional sites.

We also looked at the PANTHER protein classes to check if different functional sites were enriched in the different classes (Supplementary Figure 17 available online at http://bib.oxfordjournals.org/). Given our limited dataset, we could not compare the various properties across all the protein classes (see analysis of three PATHER classes in Supplementary Text 5 available online at http://bib.oxfordjournals.org/); however, a detailed study across all the classes has previously been done for a larger dataset of human proteins by Iqbal and coworkers [88].

### Comparison of disease-associated mutations and polymorphisms

**Figure 9:**
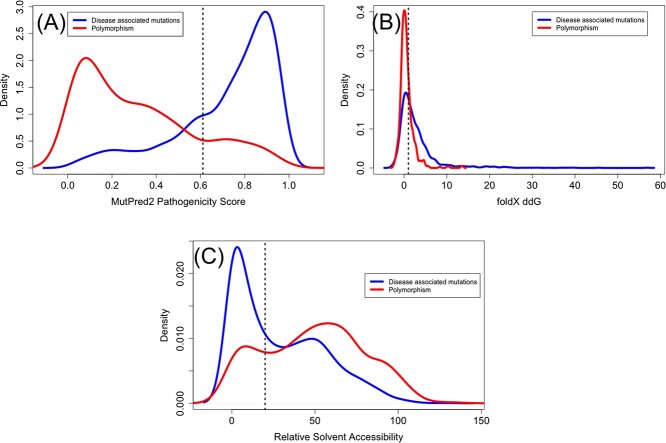
Density plots showing the distribution of (**A**) MutPred2 predicted pathogenicity scores (**B**) FoldX predicted ddG values and (**C**) relative solvent accessibility of residues that have disease-associated mutations in blue and polymorphism in red. The dotted horizontal lines show the cutoff beyond which the mutations are predicted as pathogenic (>0.611), destabilizing (>1) and buried (<20).

In order to compare the disease-associated mutations with that of likely neutral variants (polymorphisms), we checked the ddG of mutation, proximity to predicted functional sites, pathogenicity and solvent accessibility of these polymorphisms in the AlphaFold models. The number of residues with polymorphisms was 998 (see Supplementary Text 6 available online at http://bib.oxfordjournals.org/). We see that a larger percentage of disease-associated mutations were closer to the functional site, buried, destabilizing and pathogenic as compared with polymorphisms (Table 1 and Figure 9). The distribution of the pathogenicity scores, relative solvent accessibility, ddG for the polymorphisms and disease-associated mutations is significantly different (*P*-value < 2.2e-16) when compared using the Mann–Whitney Wilcox test [89]. When we compared the relative abundance of amino acids in deleterious mutations (compared with amino acids in polymorphisms), we noticed that Cys, Pro, Arg, Tyr and Trp are more predominant in deleterious mutations compared with polymorphisms (Supplementary Figure 16 available online at http://bib.oxfordjournals.org/; see Results Section on *Characterization of disease-associated mutations* for the effect of these mutations on protein function and structure).

**Table 1:** Table showing the percentage of residues that are near functional site, buried, destabilizing and pathogenic for a residue that has a disease-associated mutation compared with those residues with polymorphism

	Disease-associated mutation (%)	Polymorphism (%)
In or near predicted functional site	60	41
Destabilizing (foldX ddG > 1)	59	26
Buried (RSA < 20)	42	22
Pathogenic (MutPred2 score > 0.611)	67	15

**Figure 10:**
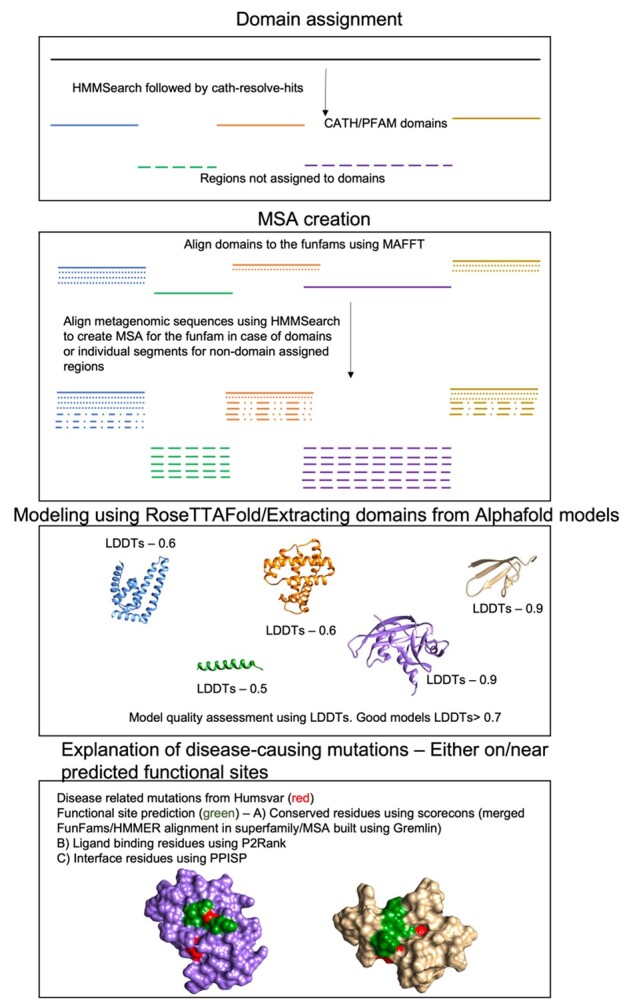
Schematic explaining the methodology to model protein domains and explain disease-associated mutations.

## DISCUSSION

With the current improved state-of-the-art computational structural predictors, like RoseTTAFold and AlphaFold, we can model regions of proteins without any known homologs for about 20% of the residues in humans [90]. In this study, we modeled the disease-associated human proteins without known homologs [calculated using Basic Local Alignment Search Tool (BLAST)] using RoseTTAFold and AlphaFold. We used these models to check if the disease-associated mutations were near predicted functional residues (i.e. ligand-binding site/protein–protein interface/conserved residues). Some of these disease-associated mutations were also predicted to be structurally destabilizing or pathogenic.

For our analyses, we used the BLAST method, which is most commonly used by biologists to detect structural homologs in the PDB. However, in principle, more sophisticated sequence search tools could be used and biologists may seek to employ MODELLER [26] or similar tools. We checked using MODELLER and this resulted in ~6% of the domains (at least 50% of the protein sequence with at least 30% sequence identity) having homologs in the PDB, giving no substantial changes to the trends (Supplementary Figure 18 and Supplementary Table 3 available online at http://bib.oxfordjournals.org/).

The AlphaFold models (72%) were largely of better quality when compared with those of RoseTTAFold models (58%). However, there were a total of 74 models where RoseTTAFold produced a good-quality model whereas AlphaFold had a low-quality model. We should keep in mind that model quality calculations for the two techniques were independent of each other and no consensus model quality assessment tool was used. To date, there has been no evaluation of the model quality estimates of AlphaFold and RoseTTAFold. However, optimizing model quality cutoffs for such consensus estimators will involve benchmarking and is out of the scope of the current study. The high-confidence models (where both the techniques provide consensus structures) could be used to predict and design inhibitors and drugs against them [91], predict off-target effects of putative drugs [92], explain the functioning of proteins, the impact of variations [93, 94] or model protein complexes [95].

The CATH domains were modeled better compared with domains that could only be assigned Pfam or not assigned to either because of lower disorder and greater diversity in the alignments used for modeling. With the ease of sequencing and large-scale sequencing projects, we expect to further increase the repertoire and quality of protein sequences in the near future. With the increased number of sequences, the model quality of the Pfam and unassigned domains will hopefully improve.

Functional sites, such as ligand-binding sites, interface residues, allosteric sites, post-translational modification sites, etc., are more conserved compared with the rest of the protein; hence mutation of these conserved sites and their neighbors might lead to a loss or modification in protein function. We considered residues close to the predicted functional site because these residues can have an effect on the structure and functioning of the functional site residues and hence changes to them might also alter the functioning of the protein. Moreover, previous studies have shown that in a large number of cases, the disease-associated mutations are near functional sites [77, 96]. We assume that the neighborhood of the functional sites will be well predicted at this level of model quality even if the functional residues are not always correct. Interface residues and ligand-binding sites have been shown to be highly conserved [97]. In our dataset, 38% of the disease-associated ligand-binding sites and 45% of the protein–protein interfaces are conserved, hence providing higher confidence that these mutations could impact structure or function. In addition, around 59% of these mutations were predicted to be structurally destabilizing (based on FoldX), which might also impact structure and function. About 85% of these disease-associated mutations in the buried residues were predicted to be destabilizing. Around 67% of the disease-associated mutations were also predicted to be pathogenic. We also had 37% of the mutations being predicted near functional sites using both AlphaFold and RoseTTAFold models, which provide additional confidence in those predictions. We were able to explain 78% of the disease-associated mutations in the good-quality regions. Given the fact that the models built using the state of art tools AlphaFold and RoseTTAFold might still have inaccuracies, cases, where the structure predicted using multiple techniques are similar provide higher confidence in the prediction accuracies. We also compared the disease-associated mutations with the polymorphisms and found a larger proportion of disease-associated mutations were more buried, destabilizing, pathogenic and had a larger percentage closer to predicted functional sites.

The fact that a large number of disease-causing mutations could be explained using the AlphaFold models further validates the model quality as we would expect poor models to introduce noise into our analyses. Predicting whether mutations are likely to be disease-associated remains one of the most challenging tasks in bioinformatics despite the improvements brought about by deep learning. This is because multiple factors can lead to a disease phenotype, such as stability of the protein, change in binding affinity, expression levels of the protein, minimum concentration of the proteins required for its functioning, etc. Our study can help in providing insights on the structural and functional features that are frequently disrupted by disease-causing mutations.

In summary, the strategy of using good-quality protein structures to predict functional sites can help explain the mechanism of disease-associated mutations. Similar strategies can be used on good-quality models of the entire human proteome in the future to explain disease-associated mutations.

## Methods

### Dataset of disease-associated human proteins to model

#### Curation of disease-associated human proteins

The VarSite database [53] contains 4444 human proteins that are disease associated as annotated in Uniprot or ClinVar. Out of these, 553 human disease proteins are without any structural homologs (match determined by running the sequence with BLAST [98] against all PDB [52] sequences with e-value cutoff of 1e-06). We used PANTHER to study the GO enrichment of these proteins. In addition, we divided the proteins into the 24 PANTHER classes (Supplementary Table 2 available online at http://bib.oxfordjournals.org/) to study the properties of each group.

#### Assignment of domain boundaries to disease-associated human proteins without structural homologs

All the 553 proteins were scanned against CATH-FunFams v4.3 (212872 HMMs) using HMMER3 [99] with an e-value cutoff of 1e-03 to assign domains. The domain boundaries were resolved using cath-resolve-hits [100] with a bitscore cutoff of 25 and coverage of 80%. The proteins/regions of proteins that were not assigned to CATH domain boundaries were scanned against the Pfam32-FunFams (102712 HMMs) using the same protocol. The regions not belonging to domains identified by scans against CATH/Pfam FunFams were modeled separately. These regions have been referred to as unassigned domains.

### Modeling the proteins using RoseTTAFold

#### MSA generation for protein domains

The domains that were assigned to the CATH/Pfam FunFams were aligned to the FunFams using MAFFT (version 7.471) [101] using the default options. The resulting seed alignments were enriched with additional homologs by performing iterative sequence hhblits [102] searches against uniclust30 (version UniRef30_2020_01) [103] and BFD [104, 105] databases as outlined by Anishchenko and colleagues [106] (details in Supplementary Text 12 available online at http://bib.oxfordjournals.org/).

#### Assessment of diversity of MSA

The diversity of the alignment was accessed using four parameters: Neff, DOPs, percent scorecons and number of taxon IDs in the alignment (Details in Supplementary Text 11 available online at http://bib.oxfordjournals.org/). DOPs and scorecons were calculated using Cathy.

#### Modeling of the protein domains

Using MSAs from Section on *MSA generation for protein domains* above, structure templates were identified by hhsearch [102] searches against the PDB100 database and 10 best scoring templates were selected. The identified templates along with the MSA were then used as inputs to RoseTTAFold [36] to predict residue–residue distances and orientations followed by the PyRosetta-based [107] 3D structure reconstruction protocol from trRosetta [33]. About 15 structure models were generated and subsequently rescored according to lDDT scores [66] predicted by DeepAccNet [108]. As revealed by the results of the CASP14 experiment [106], rescoring the pool of the PyRosetta-generated models with DeepAccNet brings in an additional 1–2 GDT_TS score [109] unit improvement in the model accuracy, which is beyond what can be achieved from picking models by Rosetta energy [110]. The model with the highest predicted global lDDT score was selected for further analysis.

#### Creating domains from AlphaFold models

The domains were extracted from the AlphaFold models downloaded from the AlphaFold database portal. The domain boundaries were the same as obtained by the CATH/Pfam scans or unassigned domains as mentioned in Section *on Assignment of domain boundaries to disease-associated human proteins without structural homologues*. The AlphaFold domains were superimposed on the domains built using RoseTTAFold using GESAMT [111].

### Scoring the local and global quality of the models

The model quality for both RoseTTAFold and AlphaFold models was calculated using the predicted lDDT scores; which is commonly used by CAPRI [66, 112] (details in Supplementary Text 11 available online at http://bib.oxfordjournals.org/). The global quality of the model is calculated as the average of the local lDDT scores of the constituent residues.

### Disorder prediction on the domain sequences

The disorder percentage was calculated for the unassigned regions using IUPred2A [68] using default parameters. A residue was considered disordered if the prediction probability was greater than 0.5.

### Prediction of functional sites on protein domains and known mutational sites

The human mutations were identified from humsavar [23] as downloaded on 2 December 2020. The mutations were classified as disease (used for disease-associated mutations), polymorphism and unclassified (refer Supplementary Text 7, available online at http://bib.oxfordjournals.org/, for current nomenclature). The conserved positions were identified as positions in the MSA with a high scorecons value (≥0.8) or position of >0.65 scorecon value near (<5 Å) a position of high scorecon value (>0.8) (details about MSA generation for identification of conserved residues are given in Supplementary Text 12 available online at http://bib.oxfordjournals.org/). The neighborhood of the predicted functional residues was identified using the implementation of the cell list algorithm [113–116] (details about choice of algorithm are given in Supplementary Text 4 available online at http://bib.oxfordjournals.org/).

The ligand-binding sites were predicted using P2Rank [117] with default parameters. Positions were annotated as ligand binding if the probability of the ligand binding was ≥0.5 (intuitively chosen). The protein–protein interaction sites were predicted using the meta-PPISP webserver [118]. The mutation was predicted to affect the functioning of the protein if the site was either on or near (within 5 Å) of a predicted functional site by P2Rank/meta-PPISP/highly conserved position. We only considered predictions for those sites where the entire domain had a good model quality score and the local quality score of the mutated residue was good (lDDT score of 70 for AlphaFold models and 0.7 for RoseTTAFold models).

We calculated the relative solvent accessibility of the residues using NACCESS [119]. For each of these sites, the effect of mutation on the stability of the protein was identified using FoldX [43]. In order to be stringent; ddG > 1 was chosen as destabilizing. In addition to FoldX, the ddG of the mutation was also calculated using DynaMut2 API [86]. This technique predicts a mutation as destabilizing if ddG < 0. The likely pathogenic impact of the mutations was predicted using the standalone version of MutPred2 [47] using default parameters (details about the tool chosen are given in Supplementary Text 4 available online at http://bib.oxfordjournals.org/). The entire method has been described schematically in Figure 10.

**Key Points**

- Recent advances in deep learning based techniques like AlphaFold and RoseTTAFold has provided near accurate protein structure models which can help explain disease associated mutations.
- The models built for CATH domains were of better quality as compared to the domains that could only be assigned to Pfam families of unknown structure, which were better than those which could not be assigned to either.
- We could explain 80% of the disease associated mutations in well modelled regions of the protein either based on proximity to predicted functional sites or destabilization of the protein or pathogenicity of the mutation.
- Usage of multiple state of art techniques for both modelling and functional sites predictions provides better confidence on the predictions.

## Supplementary Material

Supplementary_BiB_bbac187

## Data Availability

We have also tabulated all the disease-associated positions, their predicted functional site impacts, ddG of mutations, pathogenicity scores and other characteristics here (https://zenodo.org/record/6359971#.YoI6ly8w2_w). The table in the link also lists the known or predicted functions of the proteins given by the experimental GO terms for the FunFam in which the proteins are classified. All the data and the models can be accessed here (https://zenodo.org/record/6359971#.YoI6ly8w2_w).

## Author Biographies

**Neeladri Sen** is a postdoctoral fellow with Prof. Orengo, UCL working on prediction on functional sites in proteins and its application.

**Ivan Anishchenko** is an acting instructor with Prof. Baker, UW working on protein structure prediction and design.

**Nicola Bordin** is a postdoctoral fellow with Prof. Orengo, UCL whose research exploits algorithms for protein domain characterization and functional annotations. The resulting methods are applied to a variety of specific questions related to protein function and evolution.

**Ian Sillitoe** is a Principal Research Associate with Prof. Orengo, UCL who maintains and develops the CATH database and Genome3D resource.

**Sameer Velankar** leads the Protein Data Bank in Europe team and is interested in macromolecular structure archiving and in the integration of macromolecular structure data with biomedical data resources.

**David Baker** is a professor of Biochemistry at University of Washington and the director of Institute for Protein Design. His group is focused on design of macromolecular structures and functions.

**Christine Orengo** is professor of Bioinformatics at University College London. Her group develop algorithms for structure comparison/classification and for protein-function prediction, also for the analysis of functional genomics data.
